# Frailty increases the long-term risk for fall and fracture-related hospitalizations and all-cause mortality in community-dwelling older women

**DOI:** 10.1093/jbmr/zjad019

**Published:** 2024-01-04

**Authors:** Elsa Dent, Jack Dalla Via, Trent Bozanich, Emiel O Hoogendijk, Abadi K Gebre, Cassandra Smith, Kun Zhu, Richard L Prince, Joshua R Lewis, Marc Sim

**Affiliations:** Research Centre for Public Health, Equity and Human Flourishing, Torrens University Australia, Adelaide, South Australia 5000, Australia; Nutrition and Health Innovation Research Institute, School of Medical and Health Sciences, Edith Cowan University, Joondalup, Western Australia 6027, Australia; Nutrition and Health Innovation Research Institute, School of Medical and Health Sciences, Edith Cowan University, Joondalup, Western Australia 6027, Australia; Department of Epidemiology and Data Science, VU University Medical Center, Amsterdam UMC, 1081 HV, Amsterdam, Netherlands; Ageing and Later Life Research Program, Amsterdam Public Health Research Institute, Amsterdam UMC, 1081 HV, Amsterdam, Netherlands; Nutrition and Health Innovation Research Institute, School of Medical and Health Sciences, Edith Cowan University, Joondalup, Western Australia 6027, Australia; Nutrition and Health Innovation Research Institute, School of Medical and Health Sciences, Edith Cowan University, Joondalup, Western Australia 6027, Australia; Medical School, The University of Western Australia, Perth, Western Australia 6009, Australia; Australian Institute for Musculoskeletal Science (AIMSS), University of Melbourne and Western Health , St Albans, Victoria 3021, Australia; Medical School, The University of Western Australia, Perth, Western Australia 6009, Australia; Department of Endocrinology and Diabetes, Sir Charles Gairdner Hospital, Perth, Western Australia 6009, Australia; Nutrition and Health Innovation Research Institute, School of Medical and Health Sciences, Edith Cowan University, Joondalup, Western Australia 6027, Australia; Medical School, The University of Western Australia, Perth, Western Australia 6009, Australia; School of Public Health, Curtin University, Perth, Western Australia 6102, Australia; Nutrition and Health Innovation Research Institute, School of Medical and Health Sciences, Edith Cowan University, Joondalup, Western Australia 6027, Australia; Medical School, The University of Western Australia, Perth, Western Australia 6009, Australia; School of Public Health, Sydney Medical School, The University of Sydney, Hospital at Westmead, Sydney, New South Wales 2006, Australia; Nutrition and Health Innovation Research Institute, School of Medical and Health Sciences, Edith Cowan University, Joondalup, Western Australia 6027, Australia; Medical School, The University of Western Australia, Perth, Western Australia 6009, Australia

**Keywords:** injurious falls, hip fractures, cardiovascular mortality, frailty index, linked health records

## Abstract

Frailty is associated with declines in physiological capacity across sensory, neurological, and musculoskeletal systems. An underlying assumption is that the frailer an individual, the more likely they are to experience falls and fractures. We examined whether grades of frailty can assess the long-term risk of hospitalized falls, fractures, and all-cause mortality in 1261 community-dwelling older women (mean age [SD] of 75.1 [2.7] yr) over 14.5 yr. Frailty was operationalized using a frailty index (FI) of cumulative deficits from 33 variables across multiple health domains (physical, mental, comorbidities) at baseline. The total score across these variables was summed and divided by 33 to obtain the FI. Participants were graded as fit (FI ≤ 0.12), mildly frail (FI > 0.12–0.24), moderately frail (FI > 0.24–0.36), or severely frail (FI > 0.36). Fall-related (*n* = 498), any fracture-related (*n* = 347), and hip fracture–related hospitalizations (*n* = 137) and deaths (*n* = 482) were obtained from linked health records. Associations between FI grades and clinical outcomes were analyzed using multivariable-adjusted Cox-proportional hazard models including age, treatment (calcium/placebo), BMI, smoking history, socioeconomic status, plasma vitamin D (25OHD) status plus season obtained, physical activity, self-reported prevalent falls in the last 3 mo, and self-reported fractures since the age of 50 yr. At baseline, 713 (56.5%), 350 (27.8%), 163 (12.9%), and 35 (2.8%) of women were classified as fit, mildly frail, moderately frail, and severely frail, respectively. Women with mild, moderate, and severe frailty had significantly higher hazards (all *P* < .05) for a fall-related (46%, 104%, 168%), any fracture-related (88% for moderate, 193% for severe frailty), hip fracture–related hospitalizations (93%, 127%, 129%), and all-cause mortality (47%, 126%, 242%). The FI identified community-dwelling older women at risk for the most serious falls and fractures and may be incorporated into risk assessment tools to identify individuals with poorer clinical prognosis.

## Lay Summary

Frailty is often linked to decline in the sensory, neurological, and musculoskeletal systems. Frailty can be identified and graded using a frailty index (FI). In a cohort of 1261 community-dwelling older women with an average age of 75 yr, this study found that increasing grades of frailty fit, (mildly, moderately and severely frail) were associated with higher long-term risk for a fall and/or fracture (including hip fractures) that required hospitalization. Increasing grades of frailty were also linked with greater risk of death, especially due to cardiovascular causes. In conclusion, the FI may be used to identify community-dwelling older women with a high long-term risk for the most serious falls and fractures, as well as individuals with a poorer prognosis.

## Introduction

Between now and 2050, the global population of older adults (aged 65 yr and over) is projected to double, reaching over 1.5 billion people.^1^ There is a growing concern that population aging will place increased pressure on hospitals and aged care services, with many countries not yet prepared for this rapid growth in the older population.^2^^,^^3^ A major public health challenge accompanying population aging is the geriatric condition of frailty.^3^ Frailty is a clinical state characterized by an increased susceptibility to stressors, wherein if a minor stressor occurs, an individual rapidly deteriorates in health and may fail to return to their previous level of functioning.^2^^,^^3^ Compared with their non-frail counterparts, older adults living with frailty have a higher likelihood of mortality, unmet care needs, and early admission to long-term care.^2^^,^^3^ Globally, frailty is common in older adults, affecting 24% of community-dwelling older adults (as measured with the frailty index [FI] of cumulative deficits model), with this prevalence higher in women (29%) than in men (20%).^4^

Frailty is associated with declines across sensory, neurological, and musculoskeletal systems,^2^^,^^5^ which in turn increase an individual’s susceptibility to falls.^6^ Stemming from this observation is that the first clinical presentation of frailty is often a fall leading to hospitalization.^2^ In addition to hospitalization, falls often lead to disability, mortality, and a high health care burden in older populations.^6^ Approximately one-third of adults aged 65 yr and older fall annually, with 10% of these falls resulting in injury.^7^ Older women are more likely than men to have at least one fall annually or to have a fall-related injury.^7^ Furthermore, older women are more likely than older men to sustain a major osteoporotic fracture.^8-10^ Hip fractures are the most devastating fracture type in older adults^9^ and are strongly associated with mortality, long-term disability, reduced quality of life, and high health care costs.^8-10^

Emerging literature has linked frailty in older adults with an increased likelihood of osteoporotic fractures.^11^ The underlying assumption is that the more frail an individual is, the more likely they are to have an osteoporotic fracture in the future.^11^ Indeed, the high mortality rate observed in older hip fracture patients has been attributed to high frailty levels of patients prior to their falls.^9^ However, previous studies have either had relatively short follow-up periods^12^^,^^13^ or have relied on self-reported falls by study participants.^14^^,^^15^ To our knowledge, no research has examined the long-term consequences of frailty in relation to fall- and fracture-related hospitalizations. Importantly, hospitalized falls are the most serious in nature, often with resultant fractures severely impacting on an older person’s independence and quality of life.^16^

In the current study, we adopt the commonly used FI cumulative deficits model,^5^ which is commonly used across all health care settings to identify frailty.^2^ In England, all general practices are mandated to routinely identify older adults living with frailty through an electronic FI derived from patient electronic medical records, with individuals identified at risk referred for appropriate interventions.^17^ In our study, we distinguish grades of frailty from the FI (fit, mildly frail, frail, and severely frail).^5^ Limited research has investigated grades of frailty using the FI to predict falls and fractures in older adults.^11^ Moreover, comparatively very little research into long-term outcomes of frailty has been conducted in women.^18^ We investigated whether different grades of frailty can assess risk of serious adverse clinical outcomes, including fall- and fracture-related hospitalizations, as well as all-cause mortality, in community-dwelling older women over 14.5 yr, which is a longer follow-up period than most previous research on falls and fractures. A major advantage of this long-term follow-up period is that it enables earlier identification of those at risk of falls and fractures and in turn guides preventative strategies.

## Methods

### Participants

In 1998, the Calcium Intake Fracture Outcome Study (CAIFOS) began, which was a randomized controlled trial aimed at preventing fractures in women by providing them with daily calcium supplements for 5 yr.^19^ A total of 1500 women who were at least 70 yr old, expected to live beyond 5 yr, and were not taking any medication that could impact bone metabolism, such as hormone replacement therapy, were recruited, as described previously.^16^^,^^20^ After CAIFOS, women were observed for a further 10 yr, with the entire study known as the Perth Longitudinal Study of Ageing Women (PLSAW). The FI was unable to be calculated in 83 women due to missing one or more variables as part of the 33-items needed at baseline. A further 22 women taking vitamin D supplements, with an additional 103 women with missing data for circulating plasma 25OHD were excluded due to its link with falls^16^ and fracture^20^ in this cohort. Women with additional missing covariates were also excluded (*n* = 31). The current study included 1261 women (Supplementary Figure 1). All women involved in the study provided written informed consent, and ethical approval was granted by the Human Ethics Committee of the University of Western Australia. Both CAIFOS and PLSAW followed the guidelines of the Declaration of Helsinki and were registered retrospectively on the Australian New Zealand Clinical Trials Registry (ACTRN #12615000750583 and #2617000640303). Approval for linked data ethics was obtained from the Human Research Ethics Committee of the Western Australian Department of Health (#2009/24). STROBE guidelines for observational studies were also adhered to throughout the research.

### Frailty index

To construct the Rockwood FI, we followed the standard procedure described by Searle et al.^21^ who proposed criteria requiring at least 30 variables across multiple health domains; disability in activities of daily living (ADLs), instrumental ADLs, restricted activity, physical performance (eg, impaired walking, impaired grip strength) and general cognition, depression/mood, self-rated health, and comorbidity. The FI we developed for our study comprised 33 items across health domains sourced from questionnaires such as the Short form-36 and Barthel index, as well as objective measures of body composition, grip strength, timed-up-and-go performance, and blood pressure. Prevalent disease states (coronary heart disease, chronic heart failure, cerebrovascular disease, cancer, arthritis, and chronic lung disease) were determined using primary discharge diagnoses from hospital records over the previous 18 yr (1980-1998) obtained from the Western Australian Data Linkage System (Department of Health Western Australia, East Perth, Australia) and the Western Australia Hospital Morbidity Data Collection (HMDC). The ICD-coded diagnosis data were used for each different disease state and pertained to all inpatient admissions (public and private) in Western Australia. Self-reported previous medical history and medication use (eg, insulin or oral hypoglycemic agents) at baseline were used to assess prevalent diabetes mellitus. This was verified by participants’ general practitioners where possible and coded (T89001–T90009) using the International Classification of Primary Care-Plus method, which allows the aggregation of different terms for similar pathologic entities as defined by the ICD-10 coding system.^22^ A detailed list of the 33 selected variables and relevant scoring criteria is presented in Supplementary Table 1. Briefly, each variable was coded with “1” indicating the presence of the health variable deficit or “0” indicating the absence of the health variable deficit. The total score across these variables were summed and divided by 33 to obtain the FI. Women were categorized as fit (FI ≤ 0.12), mildly frail (FI > 0.12–0.24), frail (FI > 0.24–0.36), and severely frail (FI > 0.36) based on previously validated FI categories.^17^

### Clinical outcomes

Over 14.5 yr, fall-related hospitalizations, any fracture-related hospitalizations, hip fracture–related hospitalizations, and all-cause mortality were obtained using linked health records. As part of additional analysis, 5-yr self-reported falls and clinical fractures were also considered.

### Incident fall- and fracture-related hospitalizations and mortality over 14.5 yr

The Western Australian Data Linkage System and the Western Australia HMDC were used to retrieve this information, starting from the baseline clinical visit of each participant in 1998 to 2013. Diagnosis codes were defined using the International Classification of Diseases, Injuries, and Causes of Death: Clinical Modification (ICD-9-CM)^23^ codes for 1998 to 1999 that were mapped to the ICD-10 Australian Modification (ICD-10-AM)^24^ for 1999 to 2013. This included all inpatient admissions (public and private) in Western Australia. Consequently, only falls and fractures that resulted in hospitalization, often the most serious in nature, were considered here. Ascertainment of clinical outcomes from hospitalization records avoids the problems of patient self-reporting and loss to follow-up. Falls were considered injurious if hospitalization was required, and falls from standing height or less, not resulting from external force, were included (ICD-10 codes W01, W05-W08, W10, W18, and W19). Fracture identification codes included S02–S92, M80, T02, T08, T10, T12, and T14.2. Other codes pertaining to fractures of the face (S02.2–S02.6), fingers (S62.5–S62.7), and toes (S92.4–S92.5), as well as fractures caused by motor vehicle injuries were excluded (external cause of injury codes V00–V99). Primary cause of death data was used for cardiovascular disease (CVD) death (ICD-9-CM codes 390–459 and ICD-10-AM codes I00-I99), cancer death (ICD-9-CM code 140–239 excluding 210–229 and ICD-10-AM code C00-D48 excluding D10–D36), and other deaths (all other codes). Primary cause of death diagnosis text field of the death certificate was used where coded data were not yet available to ascertain the cause of death.

### Incident self-reported falls and clinical fractures over 5 yr

Self-reported falls and fractures were obtained every 4 mo over the first 5 yr as part of the CAIFOS trial. This was obtained by providing participants with a diary that they were asked to complete at the time of the adverse event including those resulting in attendance to a health care professional. Participants mailed this dairy to the research team every 4 mo before the telephone call to clarify these adverse events, which was subsequently returned to participants, as described previously.^19^ Falls were defined as unintentionally coming to rest on the ground, floor, or other lower level. For self-reported fractures, low trauma fractures resulting from falling from standing height or less, including minimally traumatic symptomatic vertebral fracture, were recorded (clinical fractures). All fractures were confirmed by radiographic records or General Practitioner reports. This method was adopted to limit recall bias while enabling “time to event” to be calculated.

### Baseline characteristics

Participants completed questionnaires regarding their smoking history and physical activity. The questionnaires assessed whether they participated in sport, recreation, or other regular physical activities in the 3 mo before the baseline visit. To calculate physical activity (kJ d^−1^), the type of activity, the duration, and the participant’s body weight were taken into account.^25^ An individual who had smoked more than one cigarette per day for more than 3 mo at any point in their life was classified as either a current smoker or an ex-smoker.

BMI (kg/m^2^) was determined by measuring body weight using digital scales and height using a stadiometer. The administration of placebo or calcium during CAIFOS was included as a covariate. Self-reported prevalent osteoporotic fractures at baseline were included if the fracture occurred after 50 yr of age, were associated with minimal trauma (falling from standing height or less), and not a fracture of the face, skull, or phalanges. Self-reported prevalent falls in the 3 mo prior to the baseline clinical visit were captured via questionnaire. The Socioeconomic Indexes for Areas developed by the Australian Bureau of Statistics were used to calculate the socioeconomic position (SEP), which ranked residential postcodes based on their relative socioeconomic advantage and disadvantage, as adopted previously.^16^^,^^20^ A higher score on the index indicates that the area has a higher proportion of families on high income and reflects levels of education, occupation, and economic status. Based on their residential postcodes, women were then classified into six predefined categories.^26^ The groups included (1) top 10% most disadvantaged, (2) highly disadvantaged (10%–30%), (3) moderate-highly disadvantaged (30%–50%), (4) low-moderately disadvantaged (50%–70%), (5) low disadvantage (70%–90%), and (6) top 10% least disadvantaged.

### Biochemistry

Baseline venous blood samples (plasma) were collected in the morning (between 0830 and 1030 h) after an overnight fast and stored at −80 °C. Total plasma 25OHD concentration for each individual was determined using a validated liquid chromatography tandem mass spectrometry method at the RDDT Laboratories, which measured plasma 25OHD_2_ and 25OHD_3_, as described previously.^16^^,^^20^ The values of 25OHD_2_ and 25OHD_3_ were summed to calculate the total plasma 25OHD concentration. The coefficients of variation were 10.1% at a mean concentration of 12 nmol L^−1^ for 25OHD_2_ and 11.3% at a mean concentration of 60 nmol L^−1^ for 25OHD_3_. Noteworthy, 1 nmol L^−1^ of 25OHD is equivalent to 0.4 ng mL^−1^. The season when the blood sample was collected (Summer [December to February], Autumn [March to May], Winter [June to August], and Spring [September to November]) was combined into two groups for descriptive purposes, Summer/Autumn vs Winter/Spring.

### Statistical analysis

Stata (V14 StataCorp LLC), IBM SPSS (V25.0, IBM Corp.), and R (V3.4.2, R Foundation for Statistical Computing)^27^ were used for all analysis. First, we examined associations between the FI and the first hospitalization for each of the clinical outcomes (fall-, any fracture-, hip fracture–related hospitalizations) and mortality using separate Cox-proportional hazard models. To allow associations to be nonlinear, restricted cubic splines within these models were used to examine the aforementioned relationships using the “rms” R package.^28^ Hazard ratio (HRs) estimates were relative to a reference value being the median FI score of fit women (FI ≤ 0.12, median 0.06) being plotted against the respective outcomes with 95% confidence bands provided. Wald tests were used to obtain *P*-values. For visual simplicity only, the *x*-axis was truncated at 3 SD above the mean. Figures were presented graphically using the “effects” R package.^29^ Global tests indicated that proportional hazards assumptions were not violated when considering the relationship between the FI, any fracture- and hip fracture–related hospitalizations, as well as all-cause mortality over 14.5 yr (*P* > .05). However, for 14.5-yr fall-related hospitalizations, the proportional hazards assumption was slightly violated (*P* = .027). We therefore truncated the data to 13.5 yr of follow-up, and no further violations of the assumptions were detected (*P* = .251). The 13.5-yr analysis for fall-related hospitalizations is now presented within Supplementary Material. Two models of adjustment were used for all analyses; minimally adjusted: age, treatment code (placebo/calcium), and BMI; and multivariable adjusted: minimally adjusted model plus physical activity, smoking history, previous fall, previous fracture, socioeconomic position, 25OHD, and season.

### Additional analysis

Fall- and fracture-related hospitalizations are likely to capture only the most serious falls and fractures. For example, falls or fractures potentially of a less severe nature that did not result in hospitalization would have been missed. As such, we also undertook analysis where we examined the relationship between FI and self-reported incident (1) falls and (2) clinical fractures over the first 5 yr in separate analysis.

Across several FI measures, older people accumulate an average deficit of 0.03 per yr.^5^ As such, we undertook survival analysis considering a 0.03 increase in FI with all four of the primary outcomes (fall-, any fracture-, hip fracture–related hospitalizations, and all-cause mortality). We also observed a positive relationship between FI and all-cause mortality. To further explore the relationship between FI and mortality, we undertook analyses between FI and various primary causes of death including cardiovascular, cancer, and all other mortality in separate analyses. For these additional analyses, we used an alternative 3-category FI proposed by Rockwood and colleagues, using FI cut-off points of <0.20, 0.20 to <0.25, and ≥ 0.25 to represent “robust”, “pre-frail,” and “frail” individuals, respectively.^5^ We stratified women into these categories and re-ran survival analysis with the primary clinical outcomes.

## Results

Baseline characteristics of participants are displayed in Table 1 with the distribution of the FI presented in Supplementary Figure 2. At baseline, 713 (56.5%), 350 (27.8%), 163 (12.9%), and 35 (2.8%) women were classified as fit, moderately frail, frail, and severely frail, respectively. Women presenting with severe frailty tended to be older, have a higher BMI, were more likely to live in a lower SEP area, and performed less physical activity, compared to fit women. Severely frail women were also more likely to present with a prevalent fall or fracture compared to fit women.

**Table 1:** Baseline characteristics stratified by frailty index (FI) categories.

**Demographics**	**All participants**	**Fit (FI ≤ 0.12)**	**Mild frailty (FI > 0.12–0.24)**	**Moderate frailty (FI > 0.24–0.36)**	**Severe frailty (FI > 0.36)**
Number	1261	713 (56.5)	350 (27.8)	163 (12.9)	35 (2.8)
Age, yr	75.1 (2.7)	**74.7 (2.6)**	**75.5 (2.7)**	**75.9 (3.0)**	**75.3 (2.9)**
Body mass index (BMI), kg m^−2^	27.1 (4.7)	**26.3 (4.1)**	**27.4 (4.8)**	**28.7 (5.4)**	**30.5 (7.1)**
Randomization					
Calcium, yes (%)	655 (51.9)	378 (53.0)	1770 (48.6)	90 (55.2)	17 (48.6)
Socioeconomic position					
Top 10% most highly disadvantaged, yes (%)	51 (4.0)	**24 (3.4)**	**14 (4.0)**	**8 (4.9)**	**5 (14.3)**
Highly disadvantaged, yes (%)	153 (12.1)	**78 (10.9)**	**49 (14.0)**	**22 (13.5)**	**4 (11.4)**
Moderate-highly disadvantaged, yes (%)	204 (16.2)	**114 (16.0)**	**54 (15.4)**	**28 (17.2)**	**8 (22.9)**
Low-moderately disadvantaged, yes (%)	184 (14.6)	**89 (12.5)**	**61 (17.4)**	**33 (20.2)**	**1 (2.9)**
Low disadvantaged, yes (%)	279 (22.1)	**166 (23.3)**	**70 (20.0)**	**34 (20.9)**	**9 (25.7)**
Top 10% least disadvantaged, yes (%)	390 (30.9)	**242 (33.9)**	**102 (29.1)**	**38 (23.3)**	**8 (22.9)**
Smoker ever, yes (%)	468 (37.1)	255 (35.8)	137 (39.1)	63 (38.7)	13 (37.1)
Physical activity, kJ d^−1^	598 (657)	**695 (662)**	**502 (598)**	**461 (719)**	**221 (370)**
Plasma 25OHD					
<50 nmol L^−1^, yes (%)	365 (28.9)	191 (26.8)	107 (30.6)	54 (33.1)	13 (37.1)
50–75 nmol L^−1^, yes (%)	462 (36.6)	254 (35.6)	133 (38.0)	63 (38.7)	12 (34.3)
≥75 nmol L^−1^, yes (%)	434 (34.4)	268 (37.6)	110 (31.4)	46 (28.2)	10 (28.6)
Season blood sample taken					
Winter/spring, yes (%)	948 (75.2)	551 (77.3)	252 (72.0)	120 (73.6)	25 (71.4)
Summer/autumn, yes (%)	313 (24.8)	162 (22.7)	98 (28.0)	43 (26.4)	10 (28.6)
Prevalent fracture from age 50 yr, yes (%)	343 (27.2)	**203 (28.5)**	**89 (25.4)**	**35 (21.5)**	**16 (45.7)**
Prevalent fall in last 3 mon, yes (%)	152 (12.1)	**59 (8.3)**	**44 (12.6)**	**37 (22.7)**	**12 (34.3)**

Data expressed as mean (SD) or number (%). Bolded values represent significant differences (*P*-value <.05) between frailty categories using ANOVA or chi-square test where appropriate.

### Frailty index and clinical outcomes from linked health data records

**Figure 1:**
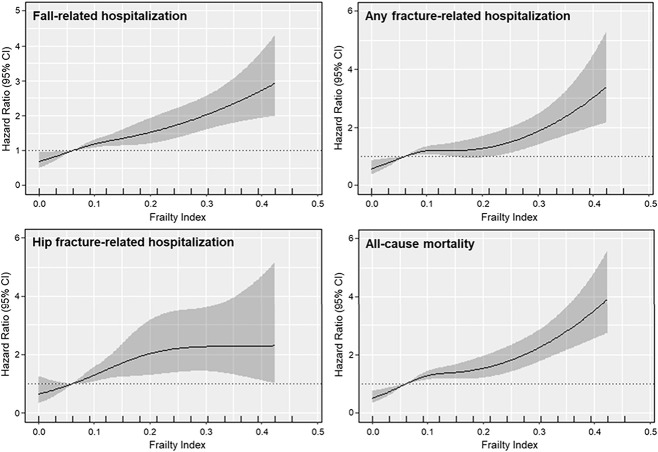
Multivariable-adjusted hazard ratios from Cox proportional hazards model with restricted cubic spline curves describing the association between the frailty index, fall-, any fracture-, and hip fracture–related hospitalizations as well as all-cause mortality over 14.5 yr. The reference is set at the median frailty index score for fit women (0.06).

Over 14.5 yr, mean follow-up was 10.9 ± 4.2 yr for a fall-related hospitalization (13 754 person years), 11.3 ± 4.1 yr for any fracture-related hospitalization (14 283 person years), 12.2 ± 3.6 yr for a hip fracture–related hospitalization (15 382 person years), and 12.6 ± 3.3 yr for all-cause mortality (15 856 person years). A diagrammatic representation for the near-linear relationships (all *P* for nonlinearity >.05) between FI and clinical outcomes is presented in Figure 1. In multivariable-adjusted models, compared to fit women, those presenting with mild, moderate, and severe frailty had 46%, 104%, and 168% greater hazards, respectively, for a fall-related hospitalization (*P* < .001, *P* for nonlinearity = .493). When follow-up for a fall-related hospitalization was truncated to 13.5 yr (mean follow-up was 10.5 ± 3.8 yr; 13 232 person years), results remained similar (Supplementary Table 2**,** Supplementary Figure 3). Specifically, women presenting with mild, moderate, and severe frailty had 51%, 113%, and 178% greater hazards, respectively, for a fall-related hospitalization, compared to fit women (*P* < .001, *P* for nonlinearity = .528). Women also had 93%, 127%, and 129% higher hazards for a hip fracture (*P* = .001, *P* for nonlinearity = .208) over 14.5 yr. For all-cause mortality, these women also recorded higher hazards of 47%, 126%, and 242%, compared to fit women (*P* < .001, *P* for non-linearity = .074). For any fracture-related hospitalization, women with moderate and severe frailty had 88% and 193% greater hazard, respectively, compared to fit women (*P* < .001, *P* for non-linearity = 0.133); however, the HR for women with mild frailty did not reach significance (HR 1.23, 95% CI 0.95–1.61) (Table 2).

**Table 2:** Hazard ratios (HR) for a fall-, any fracture- or hip fracture–related hospitalization, and all-cause mortality risk over 14.5 yr by frailty index classification.

	**Events (%)**	**Minimally adjusted**	**Multivariable adjusted**
		**HR (95% CI)**	**HR (95% CI)**
*Fall-related hospitalization*			
Fit	241/713 (33.8)	1 (reference)	1 (reference)
Mild frailty	157/350 (44.9)	**1.44 (1.16–1.80)**	**1.46 (1.17–1.82)**
Moderate frailty	82/163 (50.3)	**2.04 (1.63–2.57)**	**2.04 (1.61–2.59)**
Severe frailty	18/35 (51.4)	**2.70 (1.96–3.73)**	**2.68 (1.92–3.74)**
*Any fracture-related hospitalization*			
Fit	171/713 (24.0)	1 (reference)	1 (reference)
Mild frailty	107/350 (30.6)	1.23 (0.95–1.60)	1.23 (0.95–1.61)
Moderate frailty	53/163 (32.5)	**1.88 (1.43–2.47)**	**1.88 (1.42–2.50)**
Severe frailty	16/35 (45.7)	**2.90 (1.99–4.22)**	**2.93 (1.99–4.32)**
*Hip fracture–related hospitalization*			
Fit	61/713 (8.6)	1 (reference)	1 (reference)
Mild frailty	49/350 (14.0)	**1.94 (1.28–2.93)**	**1.93 (1.27–2.93)**
Moderate frailty	24/163 (14.7)	**2.26 (1.44–3.53)**	**2.27 (1.43–3.61)**
Severe frailty	3/35 (8.6)	**2.23 (1.14–4.36)**	**2.29 (1.14–4.59)**
*All-cause mortality*			
Fit	206/713 (28.9)	1 (reference)	1 (reference)
Mild frailty	154/350 (44.0)	**1.52 (1.22–1.90)**	**1.47 (1.17–1.84)**
Moderate frailty	98/163 (60.1)	**2.36 (1.87–2.98)**	**2.26 (1.78–2.88)**
Severe frailty	24/35 (68.6)	**3.55 (2.64–4.77)**	**3.42 (2.50–4.67)**

Frailty index (FI) categorized as fit (FI ≤ 0.12), mild frailty (FI > 0.12–0.24), moderate frailty (FI > 0.24–0.36), and severe frailty (FI > 0.36). Estimated HR and 95% CI comparing the median FI score for women classified as mildly frail (0.18), frail (0.30), and severely frail (0.39) to fit women (0.06). Minimally adjusted: adjusted for age, treatment, and BMI. Multivariable adjusted: minimally adjusted model plus smoked ever, socioeconomic status, plasma 25OHD, season of blood sampling, physical activity, self-reported prevalent falls, and prevalent fractures. Bolded values represent significant differences.

### Additional analysis

Over 5 yr, mean follow-up was 4.2 ± 1.4 yr for a self-reported fall (5332 person years) and 4.2 ± 1.5 yr for a clinical fracture (5282 person years). A diagrammatic representation for the near-linear relationships (all *P* for nonlinearity >.05) between FI and the self-reported outcomes is presented in Supplementary Figure 4. In multivariable-adjusted models, compared to fit women, those presenting with mild, moderate, and severe frailty had 31%, 80%, and 129% greater hazards, respectively, for a self-reported fall (Supplementary Table 3). For self-reported fractures, only severely frail women recorded a 90% higher hazard compared to fit women.

In multivariable-adjusted analysis, every 0.03 increase in FI was associated with greater hazards (all *P* < .001) for a fall-related (HR 1.10; 95% CI, 1.07–1.13), any fracture-related (HR 1.10; 95% CI, 1.06–1.13), hip fracture–related hospitalization (HR 1.11; 95% CI, 1.05–1.16), and all-cause mortality (HR 1.12; 95% CI, 1.09–1.15). Similar hazards were recorded for fall-related hospitalization over 13.5 yr (HR 1.10; 95% CI, 1.07–1.13). Over 14.5 yr of follow-up, 190 (15.1%), 137 (10.9%), and 155 (12.3%) women died from CVD, cancer, or any other cause, respectively. In multivariable-adjusted models, compared to fit women, those with moderate and severe frailty had 123% and 252% higher hazards for a CVD-related death, respectively (*P* < .001, *P* for nonlinearity = .265), while the hazard for those with mild frailty did not reach significance (HR 1.42; 95% CI, 0.99–2.03). No such relationship was observed for cancer-related mortality (*P* = .268, *P* for nonlinearity = .401). For all other mortality, women with mild, moderate, and severe frailty had 104%, 283%, and 544% higher hazards, respectively, compared to fit women (*P* < .001, *P* for nonlinearity = .513) (Supplementary Figure 5, Supplementary Table 4). When women were grouped into the three-category FI (robust, pre-frail, and frail) used by Rockwood and colleagues,^5^ comparable results to our four-group analysis were reported. Specifically, pre-frail and frail women had greater hazards for a fall-related (37%, 77%), fracture-related (61% for frail only), hip fracture–related hospitalization (70%, 85%) or all-cause mortality (29%, 83%) compared to robust women (*P* for non-linearity >.05 for all analysis) (Supplementary Table 5).

## Discussion

This long-term prospective study of community-dwelling older Australian women demonstrated that grades of frailty independently predicted risk of hospitalized falls, any fracture and hip fracture, self-reported falls and fractures, and all-cause mortality. To our knowledge, this is the first time the link between frailty, falls, and fractures has been reported using linked data systems using hospital-based data, in addition to self-report. Our findings thus support the hypothesis that different grades of frailty (as identified by the FI) can predict risk of serious outcomes in community-dwelling older women.

For falls and fracture outcomes, our findings are supported by previous longitudinal research. A meta-analysis of seven longitudinal studies (with a relatively short follow-up period ranging from 1 to 4 yr) reported that frailty was significantly associated with a higher risk for incident falls in both men and women, although pre-frailty did not reach statistical significance for those studies reporting HRs.^30^ Similarly, frailty and pre-frailty were both found to associate with higher risk of fractures in a meta-analysis of six studies of both men and women.^12^ However, all studies included in these systematic reviews were based on physical frailty. Although physical frailty scores are valuable tools, particularly in community settings where medical records are unavailable, they may not fully capture the multidimensional nature of frailty when compared with the FI,^2^^,^^5^ which we used in our study. The FI is a comprehensive measure of frailty that encompasses a multidimensional array of factors such as physical functioning, disability in ADL and instrumental ADL, comorbidities, memory, psychosocial factors, and self-rated health.^5^^,^^21^ It is a valid and reliable measure of frailty that is commonly used across all health care settings. The FI is especially useful in both primary care and population health studies where medical records are available, due to its objectivity and sensitivity to change.^5^^,^^21^ Importantly, the FI allows for health care providers to identify frailty in its early stages and in turn guide management interventions.^2^^,^^3^

A recent investigation in the Canadian Longitudinal Study on Aging^13^ found that the FI was associated with self-reported incident fractures over 3 yr (in both men and women aged 65 yr) and self-reported falls in the 12 mo prior to baseline (in those aged 45 yr). Yet, like most previous research, their study only considered self-reported falls and fractures. Although self-reported outcomes are clinically meaningful, they can be influenced by poor recall bias and under-reporting in older adults.^31^ To overcome limitations associated with using self-reported data alone, our study used both hospital record data and self-reported information to enhance confidence in our findings.

Women are at high risk of losing substantial BMD after menopause, and as a result, can develop osteoporosis, which is a condition characterized by weak and brittle bones that greatly increases the risk of osteoporotic fractures.^6^^,^^10^ Yet, very few long-term studies of frailty with outcomes of falls and fractures have been conducted exclusively in women. Ensrud and colleagues studied the association of physical frailty with adverse long-term outcomes in the Study of Osteoporotic Fractures and found that frailty was independently associated with increased risk of recurrent falls, hip fracture, any non-spine fracture, and mortality in women aged 70 yr and over.^14^^,^^32^ However, their study also collected self-reported data on falls, while including participants with 1 or 2 missing physical frailty domains (out of a total of 5 domains), which may have resulted in the misclassification of frailty.^14^ Similarly, Bartosch and colleagues reported that the FI was associated with recurrent self-reported falls in women aged 75 yr and over (10-yr follow-up) in their analysis of the European Osteoporosis Prospective Risk Assessment (OPRA) study.^18^ Importantly, an additional study using the OPRA dataset reported that women with frailty were at short-term risk of verified hip or major fracture (by X-rays with 2-yr follow-up), regardless of BMD.^33^ The FI has also been reported to predict risk of fractures with similar accuracy to that of the Fracture Risk Assessment Tool (FRAX), with addition of the FI to the FRAX score slightly improving fracture prediction.^34^ Importantly, this study showed that the FI was able to identify older women with high hip fracture risk who would otherwise not be prioritized for intervention.^34^ Notably, frailty was common in our study, with 15.7% of women either frail or severely frail (classified using a FI cut-off point of >0.24), and 27.8% were mildly frail (FI > 0.12–0.24). This prevalence is in line with a meta-analysis including older women of around 29%.^4^

In keeping with previous research of older women,^14^ the present study found that the FI was highly predictive of all-cause mortality. Compared to fit women, all-cause mortality during follow-up was twice as likely for those who were moderately frail and three times as likely for those who were severely frail. We also found that women with moderate and severe frailty (although not mild frailty) were at a significantly higher risk for CVD-related death. These findings are concerning and are consistent with an emerging body of literature identifying frailty as a major risk factor for CVD, an area warranting urgent investigation.^2^^,^^3^

The association between FI and risk of fracture in women can be explained, at least partially, by the higher likelihood of falls in those with frailty.^18^ Features of frailty include low muscle strength, balance problems, and gait disorders including slowing of gait speed.^2^^,^^3^ These features are all falls risk factors,^6^ which can also be influenced by 25OHD levels. Additionally, cognitive decline and sensory deficits (eg, impaired vision or hearing), which are common in individuals with frailty, increase falls risk.^6^ As we have established a link between lower plasma 25OHD levels (<50 nmol L^−1^), fall, and fracture hospitalization risk in this cohort,^16^^,^^20^ we accounted for 25OHD levels (and season the sample was obtained) in our analyses.

Our results demonstrate the need for effective strategies to prevent frailty in older women. Strategies include early identification of frailty in older women by routine screening and case finding, addressing contributing factors such as malnutrition, low physical activity and polypharmacy, and optimizing the management of chronic conditions including CVD.^2^ Future research is needed to ascertain the optimal intervention at reducing the incidence of falls and fractures in older adults with frailty, particularly in older women. Current clinical guidelines recommend resistance-based training (strength training) to prevent and manage frailty in older adults,^2^ and regular moderate-high impact weight-bearing exercise to prevent osteoporotic fractures in older women.^8^ Ideally, exercise should be combined with optimal nutrition, which includes adequate intake of protein, calcium, and vitamin D.^6^^,^^8^^,^^10^ Additionally, there is a need for future research to focus on addressing disparities in access to frailty prevention and management interventions, including in older adults with lower SEP.^3^

The current study has several strengths, including the complete variable list for derivation of the FI (eg, no missing data across 33 variables), the long-term follow-up, and robust verification of outcomes. We also controlled for a broad variety of confounding variables, including 25OHD and lifestyle factors such as physical activity, smoking, alcohol, and calcium intake. We also used linked health data from hospitals in Western Australia, and previous research noting that these older women were unlikely to move interstate, there would have been little to no loss to follow-up.^16^ Similarly, we focused primarily on falls and fractures that resulted in hospitalization, and there would have been instances where an individual suffered a fall without injury (or nonserious injuries thus not warranting hospitalization). To account for this, we also undertook analysis considering 5-yr self-reported falls and clinically verified fractures from X-rays. This analysis would consider less serious fractures that did not result in hospitalization (eg, forearm). In comparison, the use of linked hospital records eliminates self-report bias and captures the most serious events, as it was deemed necessary by the treating physician to warrant hospitalization. Despite the strengths of our study, there were limitations. Our findings may not be extrapolative to other populations (eg, younger women, men, non-Caucasian women) where frailty prevalence may be different. Furthermore, as principal hospital discharge diagnosis codes were also used to assess prevalent diseases (eg, arthritis, chronic lung disease), it is unlikely less severe states of such diseases would have been captured. This may have resulted in an underestimation of the FI. Finally, despite accounting for a range of fall and fracture risk factors, we cannot rule out the possibility of residual confounding.

In conclusion, this study found that increasing grades of frailty derived from the FI were associated with increased risk for hospitalization due to falls, any fracture, and hip fractures, as well as mortality in community-dwelling older women. Furthermore, frailty appeared to be a major driver of CVD-related mortality in older women. Our results highlight the need for early detection and management of frailty in older women, including targeted primary prevention strategies such as diet and exercise, which are known to lower risk for cardiometabolic diseases as well as falls and fractures.

## Supplementary Material

FI_Falls_Fx_All_cause_Supplementary_Material_Revised_Final_Proof_zjad019

## Data Availability

Data are available from the authors upon reasonable request in-line with governing ethical considerations.
