# Supplementary material for: Frailty increases the long-term risk for fall and fracture-related hospitalizations and all-cause mortality in community-dwelling older women
Source: J Bone Miner Res. 2024 Jan 4;39(3):222–30. doi: 10.1093/jbmr/zjad019 (PMC11240159; doi:10.1093/jbmr/zjad019)
Supplement: FI_Falls_Fx_All_cause_Supplementary_Material_Revised_Final_Proof_zjad019 [file FI_Falls_Fx_All_cause_Supplementary_Material_Revised_Final_Proof_zjad019.docx]

**Supplementary material. Dent et al. 2023.**

**Supplementary Table 1.** Variables included in the 33-item frailty index based on variables available within the Perth Longitudinal Study of Ageing Women (PLSAW) cohort.

**Supplementary Table 2.** Hazard ratios (HR) for fall-related hospitalization risk over 13.5 years by frailty index classification.

**Supplementary Table 3.** Hazard ratios (HR) for self-reported falls and fractures over 5 years by frailty index (FI) category.

**Supplementary Table 4.** Hazard ratios (HR) for cardiovascular-disease (CVD) mortality, cancer mortality, and any other mortality by frailty index (FI) category.

**Supplementary Table 5.** Hazard ratios (HR) for falls, fracture and all-cause mortality risk by alternative frailty index categories.

**Supplementary Figure 1.** Participant flowchart.

**Supplementary Figure 2.** Distribution of the frailty index in the 1261 women.

**Supplementary Figure 3**. Hazard ratios from Cox proportional hazards model with restricted cubic spline curves describing the association between frailty index and fall-related hospitalizations over 13.5 years. The reference is set at the median frailty index score for fit women (0.06).

**Supplementary Figure 4.** Hazard ratios from Cox proportional hazards model with restricted cubic spline curves describing the association between frailty index and self-reported falls and clinically verified fractures over 5 years. The reference is set at the median frailty index score for fit women (0.06).

**Supplementary Figure 5.** Hazard ratios from Cox proportional hazards model with restricted cubic spline curves describing the association between frailty index and CVD mortality, cancer mortality and other mortality over 14.5 years. Reference is the median frailty index value of fit women (0.06).

| **Supplementary Table 1.** Variables included in the 33-item frailty index based on variables available within the Perth Longitudinal Study of Ageing Women (PLSAW) cohort. | | | | |
| --- | --- | --- | --- | --- |
| **Variable *** | **Source** | **Question** | Responses | Deficit? |
| Help bathing | Barthel Index | Q2. Bathing | No attempt | Yes = 1 |
|  |  |  | Attempts |  |
|  |  |  | Some help |  |
|  |  |  | Minimal help | No = 0 |
|  |  |  | Independent |  |
| Help dressing | Barthel index | Q6. Dressing | No attempt | Yes = 1 |
|  |  |  | Attempts |  |
|  |  |  | Some help |  |
|  |  |  | Minimal help | No = 0 |
|  |  |  | Independent |  |
| Help getting in/out of chair | Barthel index | Q9. Bed to chair transfer | No attempt | Yes = 1 |
|  |  |  | Attempts |  |
|  |  |  | Some help |  |
|  |  |  | Minimal help | No = 0 |
|  |  |  | Independent |  |
| Help walking around house | Barthel Index | Q10. Ambulation | No attempt | Yes = 1 |
|  |  |  | Attempts |  |
|  |  |  | Some help |  |
|  |  |  | Minimal help | No = 0 |
|  |  |  | Independent |  |
| Help eating | Barthel Index | Q3. Feeding | No attempt | Yes = 1 |
|  |  |  | Attempts |  |
|  |  |  | Some help |  |
|  |  |  | Minimal help | No = 0 |
|  |  |  | Independent |  |
| Help grooming | Barthel Index | Q1. Personal Hygiene | No attempt | Yes = 1 |
|  |  |  | Attempts |  |
|  |  |  | Some help |  |
|  |  |  | Minimal help | No = 0 |
|  |  |  | Independent |  |
| Help using toilet | Barthel Index | Q4. Toileting | No attempt | Yes = 1 |
|  |  |  | Attempts |  |
|  |  |  | Some help |  |
|  |  |  | Minimal help | No = 0 |
|  |  |  | Independent |  |
| Help up/down stairs | Barthel Index | Q5. Stair climbing | No attempt | Yes = 1 |
|  |  |  | Attempts |  |
|  |  |  | Some help |  |
|  |  |  | Minimal help | No = 0 |
|  |  |  | Independent |  |
| Help lifting 10 lbs | SF-36 | Q5. Lifting or carrying groceries | Yes, limited a lot | Yes = 1 |
|  |  |  | Yes, limited a little |  |
|  |  |  | No, not limited at all | No = 0 |
| Help shopping | SF-36 | Q16. Had difficulty performing the work or other activities | Yes | Yes = 1 |
|  |  |  | No | No = 0 |
| Help with housework | SF-36 | Q4. Moderate activities, such as moving table, pushing a vacuum cleaner, bowling, or playing golf | Yes, limited a lot | Yes = 1 |
|  |  |  | Yes, limited a little |  |
|  |  |  | No, not limited at all | No = 0 |
| Self-Rating of health | SF-36 | Q1. In general, would you say your health is: | Excellent | No = 0 |
|  |  |  | Very good |  |
|  |  |  | Good |  |
|  |  |  | Fair | Yes = 1 |
|  |  |  | Poor |  |
| How health has changed in last year | SF-36 | Q2. Compared to one year ago, how would you rate your health in general now? | Much better now than one  year ago | No = 0 |
|  |  |  | Somewhat better now than one year ago |  |
|  |  |  | About the same |  |
|  |  |  | Somewhat worse now than one year ago | Yes = 1 |
|  |  |  | Much worse now than one year ago |  |
| Cut down on usual activity (in last month) | SF-36 | Q13. During the past 4 weeks, have you cut down the amount of time you spent on work or other activities? | Yes | Yes = 1 |
|  |  |  | No | No = 0 |
| Walk outside | Questionnaire | Any physical activity undertaken in the last three months. | Sedentary (0 kcal/day) | Yes = 1 |
|  |  |  | Any physical activity (>0 kcal/day) | No = 0 |
| Feel everything is an effort | SF-36 | Q29. During the past 4 weeks, how often did you feel worn out? | All of the time | Yes = 1 |
|  |  |  | Most of the time |  |
|  |  |  | A good bit of the time |  |
|  |  |  | Some of the time |  |
|  |  |  | A little of the time | No = 0 |
|  |  |  | None of the time |  |
| Feel depressed | SF-36 | Q25. During the past 4 weeks, how often have you felt so down in the dumps that nothing could cheer you up? | All of the time | Yes = 1 |
|  |  |  | Most of the time |  |
|  |  |  | A good bit of the time |  |
|  |  |  | Some of the time |  |
|  |  |  | A little of the time | No = 0 |
|  |  |  | None of the time |  |
| Feel happy | SF-36 | Q30. During the past 4 weeks, how often have you been a happy person? | All of the time | No = 0 |
|  |  |  | Most of the time |  |
|  |  |  | A good bit of the time |  |
|  |  |  | Some of the time |  |
|  |  |  | A little of the time | Yes = 1 |
|  |  |  | None of the time |  |
| Feel lonely | SF-36 | Q32. During the past 4 weeks, how much of the time has your physical health or emotional problems interfered with your social activities (like visiting friends, relatives, etc.)? | All of the time | Yes = 1 |
|  |  |  | Most of the time |  |
|  |  |  | A good bit of the time |  |
|  |  |  | Some of the time |  |
|  |  |  | A little of the time | No = 0 |
|  |  |  | None of the time |  |
| Have trouble getting going | SF-36 | Q23. During the past 4 weeks, how often did you feel full of pep? | All of the time | Yes = 1 |
|  |  |  | Most of the time |  |
|  |  |  | A good bit of the time |  |
|  |  |  | Some of the time |  |
|  |  |  | A little of the time | No = 0 |
|  |  |  | None of the time |  |
| High blood pressure | Physical assessment | Hypertension (systolic blood pressure ≥140 and/or diastolic blood pressure ≥90) | Yes | Yes = 1 |
|  |  |  | No | No = 0 |
| Heart attack | Linked health data:  Primary discharge diagnoses from hospital records over the previous 18-years  (1980–1998) | Prevalent coronary heart disease | Yes | Yes = 1 |
|  |  |  | No | No = 0 |
| Heart failure |  | Prevalent heart failure | Yes | Yes = 1 |
|  |  |  | No | No = 0 |
| Stroke |  | Prevalent cerebrovascular disease | Yes | Yes = 1 |
|  |  |  | No | No = 0 |
| Cancer |  | Prevalent cancer | Yes | Yes = 1 |
|  |  |  | No | No = 0 |
| Arthritis |  | Prevalent arthritis | Yes | Yes = 1 |
|  |  |  | No | No = 0 |
| Chronic lung disease |  | Prevalent chronic lung disease/COPD | Yes | Yes = 1 |
|  |  |  | No | No = 0 |
| Diabetes | Self-reported, medication use and verified by GP reports | Prevalent diabetes | Yes | Yes = 1 |
|  |  |  | No | No = 0 |
| Cognitive impairment | Mini-Mental State Examination (MSSE) | Abbreviated mental  score sum | <8 | Yes = 1 |
|  |  |  | ≥8 | No = 0 |
| BMI | Physical assessment | Weight (kg)/height (m^2^) | <21 kg/m^2^ | Yes = 1 |
|  |  |  | ≥21 kg/m^2^ | No = 0 |
| Grip strength | Physical assessment | Grip strength dynamometer | <18 kg | Yes = 1 |
|  |  |  | ≥18 kg | No = 0 |
| Usual pace | SF-36 | Q10. Does your health limit you in walking several blocks? | Yes, limited a lot | Yes = 1 |
|  |  |  | Yes, limited a little |  |
|  |  |  | No, not limited at all | No = 0 |
| Rapid pace | Physical assessment | Timed-up-and go test | >10.2 seconds | Yes = 1 |
|  |  |  | ≤10.2 seconds | No = 0 |
| * Variable names as per Searle et al. Where the exact data was not available in the PLSAW cohort, the most appropriate equivalent was used. | | | | |

| **Supplementary Table 2.** Hazard ratios (HR) for fall-related hospitalization risk over 13.5 years by frailty index classification. | | | |  |
| --- | --- | --- | --- | --- |
|  | **Events (%)** | **Minimally-adjusted** | **Multivariable-adjusted** | |
|  |  | **HR (95%CI)** | **HR (95%CI)** | |
| Fit | 205/713 (28.8) | 1 (reference) | 1 (reference) | |
| Mild frailty | 145/350 (41.4) | **1.50 (1.19-1.89)** | **1.51 (1.20-1.91)** | |
| Moderate frailty | 75/163 (46.0) | **2.14 (1.68-2.73)** | **2.13 (1.66-2.74)** | |
| Severe frailty | 17/35 (48.6) | **2.82 (2.01-3.96)** | **2.78 (1.96-3.93)** | |

Frailty index (FI) categorised as fit (FI ≤0.12), mild frailty (FI >0.12-0.24), moderate frailty (FI >0.24-0.36) and severe frailty (FI >0.36). Estimated HR and 95%CI comparing the median FI score for women classified as mildly frail (0.18), frail (0.30) and severely frail (0.39) to fit women (0.06). Minimally-adjusted: adjusted for age, treatment and body mass index. Multivariable-adjusted: minimally adjusted model plus smoked ever, socioeconomic status, plasma 25-hydroxy vitamin D, season of blood sampling, physical activity, self-reported prevalent falls, and prevalent fractures. Bolded values represent significant differences.

| **Supplementary Table 3.** Hazard ratios (HR) for self-reported falls and fractures over 5 years by frailty index (FI) category. | | | |
| --- | --- | --- | --- |
| **Self-reported over 5 years** | **Number of events (%)** | **Minimally-adjusted** | **Multivariable-adjusted** |
|  |  | **HR (95%CI)** | **HR (95%CI)** |
| *Falls* | | | |
| Fit | 176/713 (24.7) | 1 (reference) | 1 (reference) |
| Mild frailty | 107/350 (30.6) | 1.28 (0.99-1.66) | **1.31 (1.01-1.71)** |
| Moderate frailty | 57/163 (35.0) | **1.80 (1.37-2.36)** | **1.80 (1.36-2.38)** |
| Severe frailty | 18/35(51.4) | **2.36 (1.63-3.41)** | **2.29 (1.56-3.34)** |
| *Clinical fractures* | | | |
| Fit | 108/713 (8.6) | 1 (reference) | 1 (reference) |
| Mild frailty | 57/350 (16.3) | 0.94 (0.66-1.34) | 0.95 (0.67-1.35) |
| Moderate frailty | 27/163 (16.6) | 1.36 (0.95-1.96) | 1.33 (0.91-1.94) |
| Severe frailty | 10/35 (28.6) | **2.01 (1.23-3.26)** | **1.90 (1.15-3.14)** |
| FI categorised as fit (FI ≤0.12), mildly frail (FI >0.12-0.24), frail (FI >0.24-0.36) and severe frailty (FI >0.36). Estimated HR and 95%CI comparing the median FI score for women classified as mildly frail (0.18), frail (0.30) and severely frail (0.39) to fit women (0.06). Minimally-adjusted: adjusted for age, treatment and body mass index. Multivariable-adjusted: minimally adjusted model plus smoked ever, socioeconomic status, plasma 25-hydroxy vitamin D, season of blood sampling, physical activity, self-reported prevalent falls, and prevalent fractures. Bolded values represent significant differences. | | | |

| **Supplementary Table 4.** Hazard ratios (HR) for cardiovascular-disease (CVD) mortality, cancer mortality, and any other mortality by frailty index (FI) category. | | | |
| --- | --- | --- | --- |
|  | **Number of events (%)** | **Minimally-adjusted** | **Multivariable-adjusted** |
|  |  | **HR (95%CI)** | **HR (95%CI)** |
| *CVD mortality* | | | |
| Fit | 82/713 (11.5) | 1 (reference) | 1 (reference) |
| Mild frailty | 56/350 (16.0) | **1.44 (1.01-2.06)** | 1.42 (0.99-2.03) |
| Moderate frailty | 42/163 (25.8) | **2.25 (1.56-3.26)** | **2.23 (1.52-3.28)** |
| Severe frailty | 10/35 (28.6) | **3.49 (2.17-5.59)** | **3.52 (2.14-5.78)** |
| *Cancer mortality* | | | |
| Fit | 71/713 (10.0) | 1 (reference) | 1 (reference) |
| Mild frailty | 44/350 (12.6) | 1.20 (0.79-1.83) | 1.12 (0.73-1.70) |
| Moderate frailty | 18/163 (11.0) | 1.43 (0.92-2.22) | 1.27 (0.80-2.01) |
| Severe frailty | 4/35 (11.4) | 1.73 (0.92-3.26) | 1.52 (0.78-2.93) |
| *Other mortality* | | | |
| Fit | 53/713 (7.4) | 1 (reference) | 1 (reference) |
| Mild frailty | 54/350 (15.4) | **2.08 (1.39-3.11)** | **2.04 (1.36-3.06)** |
| Moderate frailty | 38/163 (23.3) | **3.92 (2.58-5.96)** | **3.83 (2.48-5.90)** |
| Severe frailty | 10/35 (28.6) | **6.56 (3.99-10.78)** | **6.44 (3.81-10.89)** |
| FI categorised as fit (FI ≤0.12), mildly frail (FI >0.12-0.24), frail (FI >0.24-0.36) and severe frailty (FI >0.36). Estimated HR and 95%CI comparing the median FI score for women classified as mildly frail (0.18), frail (0.30) and severely frail (0.39) to fit women (0.06). Minimally-adjusted: adjusted for age, treatment and body mass index. Multivariable-adjusted: minimally-adjusted model plus smoked ever, socioeconomic status, plasma 25-hydroxy vitamin D, season of blood sampling, physical activity, self-reported prevalent falls, and prevalent fractures. Bolded values represent significant differences. | | | |

| **Supplementary Table 5.** Hazard ratios (HR) for falls, fracture and all-cause mortality risk by alternative frailty index categories. | | | |
| --- | --- | --- | --- |
|  | **Number of events (%)** | **Minimally-adjusted** | **Multivariable-adjusted** |
|  |  | **HR (95%CI)** | **HR (95%CI)** |
| *Fall-related hospitalization* | | | |
| Robust | 324/912 (35.5) | 1 (reference) | 1 (reference) |
| Pre-frail | 74/151 (49.0) | **1.38 (1.08-1.76)** | **1.37 (1.07-1.74)** |
| Frail | 100/198 (50.5) | **1.80 (1.42-2.28)** | **1.77 (1.39-2.26)** |
| *Any fracture-related hospitalization* | | | |
| Robust | 233/912 (25.5) | 1 (reference) | 1 (reference) |
| Pre-frail | 45/151 (29.8) | 1.15 (0.86-1.54) | 1.13 (0.84-1.51) |
| Frail | 69/198 (34.8) | **1.65 (1.24-2.19)** | **1.61 (1.20-2.16)** |
| *Hip fracture-related hospitalization* | | | |
| Robust | 90/912 (9.9) | 1 (reference) | 1 (reference) |
| Pre-frail | 20/151 (13.2) | **1.73 (1.10-2.75)** | **1.70 (1.07-2.70)** |
| Frail | 27/198 (13.6) | **1.86 (1.17-2.98)** | **1.85 (1.15-2.99)** |
| *All-cause mortality* | | | |
| Robust | 289/912 (31.7) | 1 (reference) | 1 (reference) |
| Pre-frail | 71/151 (47.0) | **1.33 (1.04-1.71)** | **1.29 (1.00-1.65)** |
| Frail | 122/198 (61.6) | **1.90 (1.50-2.42)** | **1.83 (1.43-2.34)** |
| FI categorised as robust (FI <0.20), pre-frail (FI 0.20-<0.25) and frail (FI ≥0.25). Estimated HR and 95%CI comparing the median FI score for women classified as pre-frail (0.22) and frail (0.31), compared to fit women (0.09). Minimally-adjusted: adjusted for age, treatment and body mass index. Multivariable-adjusted: minimally adjusted model plus smoked ever, socioeconomic status, plasma 25-hydroxy vitamin D, season of blood sampling, physical activity, self-reported prevalent falls, and prevalent fractures. Bolded values represent significant differences. | | | |


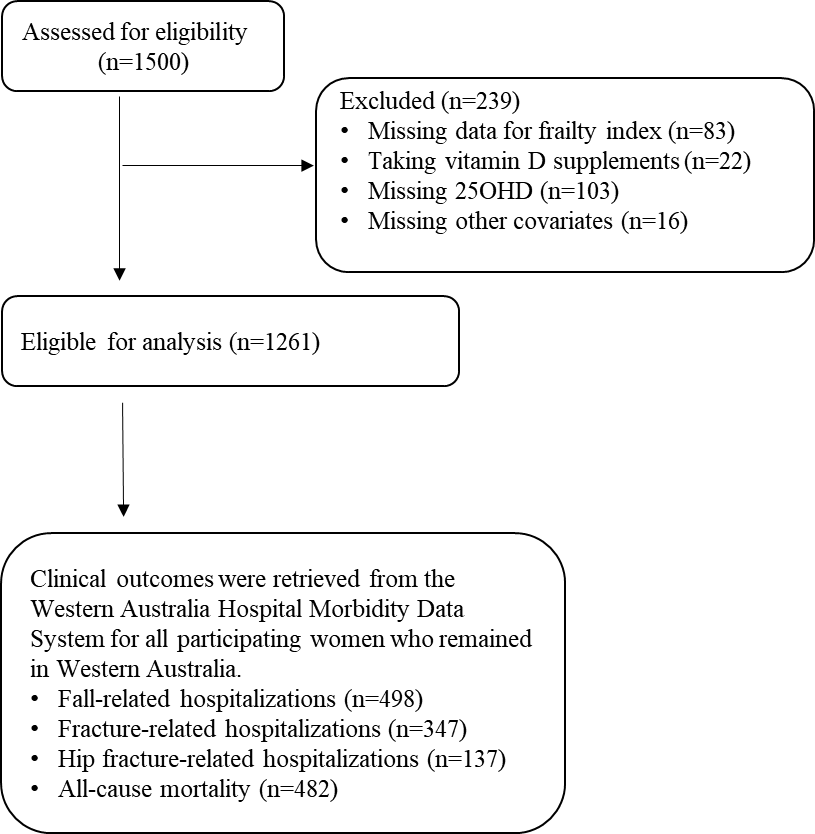


**Supplementary Figure 1.** Participant flowchart.

**
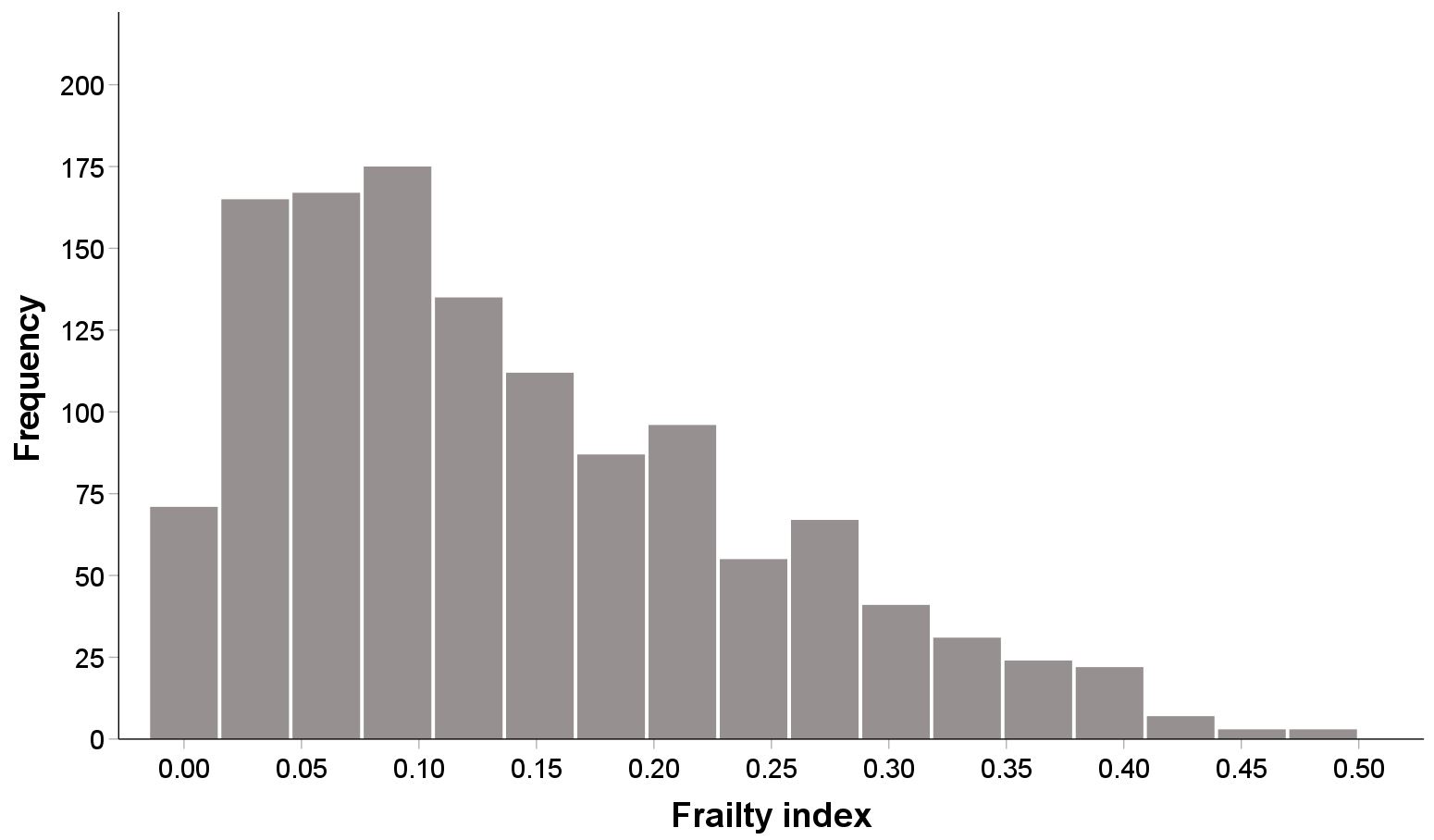
**

**Supplementary Figure 2.** Distribution of the frailty index in the 1261 women.


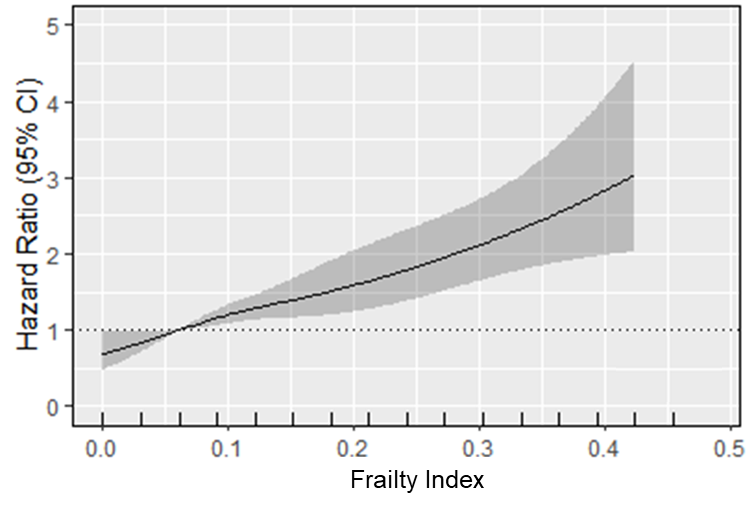


**Supplementary Figure 3**. Hazard ratios from Cox proportional hazards model with restricted cubic spline curves describing the association between frailty index and fall-related hospitalizations over 13.5 years. The reference is set at the median frailty index score for fit women (0.06).


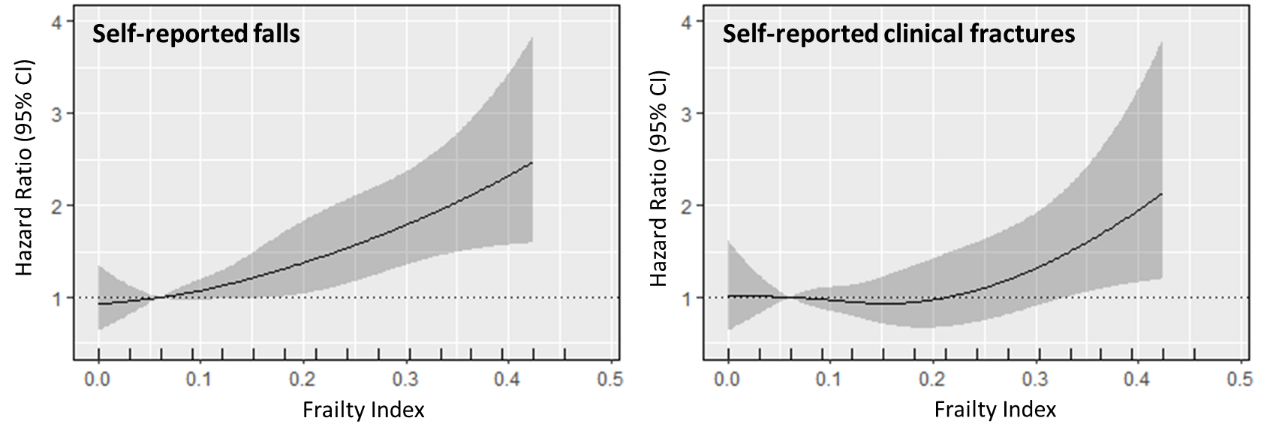


**Supplementary Figure 4.** Hazard ratios from Cox proportional hazards model with restricted cubic spline curves describing the association between frailty index and self-reported falls and clinically verified fractures over 5 years. The reference is set at the median frailty index score for fit women (0.06).

**
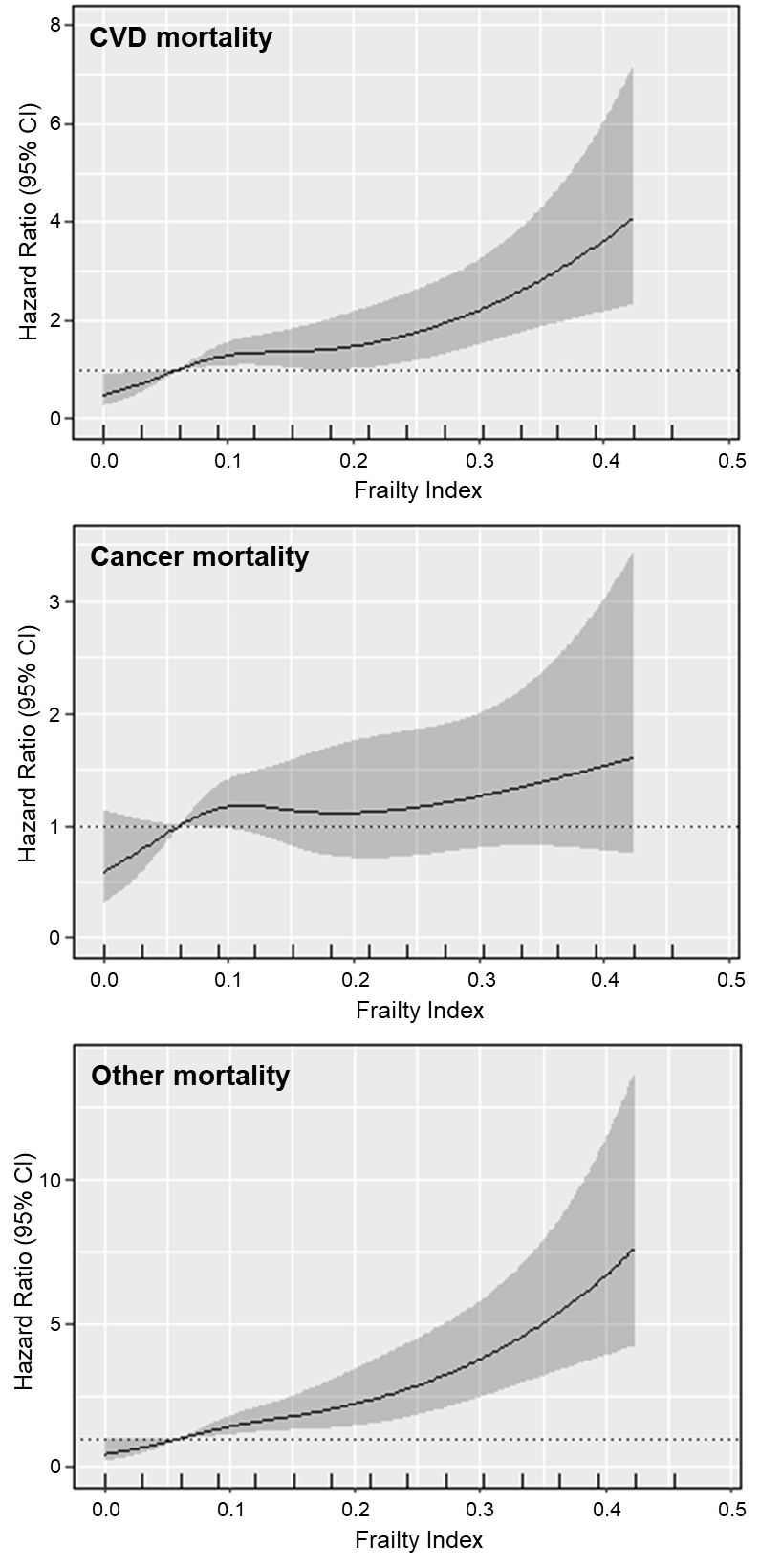
**

**Supplementary Figure 5.** Hazard ratios from Cox proportional hazards model with restricted cubic spline curves describing the association between frailty index and CVD mortality, cancer mortality and other mortality over 14.5 years. Reference is the median frailty index value of fit women (0.06).
